# Complete mitochondrial genome of white-backed woodpecker *Dendrocopos leucotos* (Piciformes: Picidae) and its phylogenetic position

**DOI:** 10.1080/23802359.2017.1357454

**Published:** 2017-07-25

**Authors:** Soo Hyung Eo

**Affiliations:** Department of Forest Resources, Kongju National University, Yesan-eup, Chungnam, Republic of Korea

**Keywords:** Avian mtDNA, gene order, mitogenome, phylogeny

## Abstract

The white-backed woodpecker (*Dendrocopos leucotos*) is an ecologically important bird in Eurasian forest ecosystems. In this study, the complete mitochondrial genome of the species was sequenced using next-generation sequencing technology. The genome was 16,838 bp in length, consisting of 13 protein coding genes, two rRNAs, 22 tRNAs, a non-coding control region and a repeat region. Phylogenetic analysis using available complete mitochondrial genomes of the Coraciimorphae supported the monophyly of the Piciformes. The complete mitochondrial genome of *D. leucotos* will be a useful genetic resource for population genetics, phylogenetic analysis and conservation of the species.

The white-backed woodpecker (*Dendrocopos leucotos* Bechstein, 1803: family Picidae) has a widespread distribution ranging across boreal, temperate or subtropical forests in Palearctic region (Del Hoyo et al. 2002). Despite the species still has a large global population size, the population is suspected to be in rapid decline in some regions such as Scandinavia (Aulén 1988). Habitat loss owing to intensive forest management and removal of dead wood is considered a major threat to the species (Fernandez and Azkona 1996; Carlson 2000). Conservation and restoration of threatened populations of the species require information on genetic diversity and structure of the populations (Frankham et al. 2002; Eo et al. 2010), but very few genetic studies have been conducted on the species (e.g. Ellegren et al. 1999). In this study, complete mitochondrial genome (mitogenome) of a white-backed woodpecker was generated and characterized for genetic information on the species conservation.

Genomic DNA of a white-backed woodpecker which was road-killed near Seoul, South Korea, was isolated using DNeasy Blood & Tissue Kit (Qiagen Korea Ltd., Seoul, South Korea). The sample is stored in the Department of Forest Resources, Kongju National University, South Korea (sample database number: ESH_A00036). DNA library was constructed and sequenced on Illumina HiSeq 2500 PE100 platform. Resulting 10.6 million sequence reads with 1.33 Gbp were assembled by SPAdes v. 3.60 (Bankevich et al. 2012). The assembled mitogenome was annotated using MITOS Web Server (Bernt et al. 2013).

The complete mitogenome sequence of *D. leucotos* (GenBank accession number: KU131555) was 16,838 bp in length, consisting of 13 protein coding genes (PCGs), 12S and 16S ribosomal RNAs (*rRNAs*), 22 transfer RNAs (*tRNAs*), a non-coding control region (CR) and a repeat region. Overall GC contents were 48% (28.1% for A, 23.9% for T, 34.1% for C and 13.9% for G). Gene order in *D. leucotos* mitogenome was the same as those reported previously for other birds in Picidae (Mindell et al. 1998), including from 5′ to 3′: *ND5-Cytb-tRNA^Thr^-CR-tRNA^Pro^-ND6-tRNA^Glu^-tRNA^Phe^-12S*, with a non-coding region between *tRNA^Glu^* and *tRNA^Phe^*. This gene order was different from those in most other birds: *ND5-Cytb-tRNA^Thr^-tRNA^Pro^-ND6-tRNA^Glu^-CR-tRNA^Phe^-12S* (Mindell et al. 1998). All PCGs initiated with ATG as the start codon, except for *COX1* (ATT), *ND3* (ATA) and *ND5* (GTG).

The phylogenetic tree constructed from 13 PCGs of the *D. leucotos* mitogenome and available Coraciimorphae birds (Piciformes, Coraciiformes, Bucerotiformes and Trogoniformes) in GenBank supported the monophyly of the Piciformes (Figure 1). Genetic difference of mitogenomes between *D. leucotos* and congeneric species *Dendrocopos major* was 4.9%. The complete mitogenome of *D. leucotos* will play a significant role in population genetics, phylogenetic analysis and conservation and management of the species.

**Figure 1.**
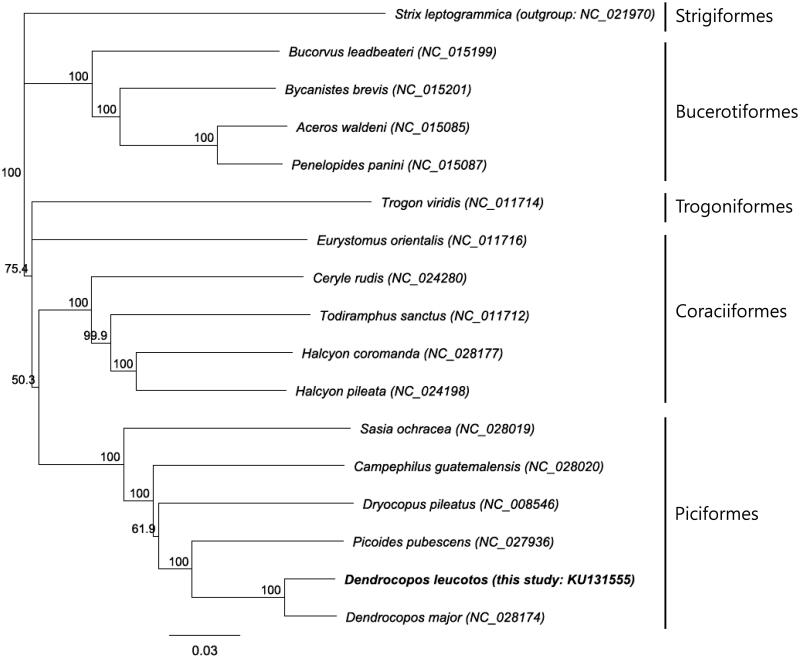
Neighbour joining tree based on 13 protein coding genes of mitogenomes of Piciformes including *D. leucotos* we sequenced (KU131555) and its related orders (Coraciiformes, Trogoniformes and Bucerotiformes). *Strix leptogrammica* (Strigiformes) was used as an out-group. Numbers on branches represent bootstrap supports (1000 replicates).
